# 

**Published:** 1969-01

**Authors:** 


					11 1
vn N r/
January 1969
NATIONAL library .of medicine
^PL-ISS;*?^ " '^C:C(H
1 DATE:\J7f// (o C?
S3 ! 'Ira ?*!? a ' Wm ^
?Ncorporating the medical journal of the south west
Vol. 84 (i) No 309
IIS
' >\v

				

